# Morphometric and Molecular Analyses of *Ostertagia leptospicularis* Assadov, 1953 from Ruminants: Species Diversity or Host Influence?

**DOI:** 10.3390/ani11010182

**Published:** 2021-01-14

**Authors:** Anna Wyrobisz-Papiewska, Jerzy Kowal, Elżbieta Łopieńska-Biernat, Paweł Nosal, Iwona Polak, Łukasz Paukszto, Steffen Rehbein

**Affiliations:** 1 Department of Zoology and Animal Welfare, Faculty of Animal Sciences, University of Agriculture in Krakow, Mickiewicza av. 24/28, 30-059 Krakow, Poland; jerzy.kowal@urk.edu.pl (J.K.); pawel.nosal@urk.edu.pl (P.N.); 2 Department of Biochemistry, Faculty of Biology and Biotechnology, University of Warmia and Mazury in Olsztyn, Oczapowskiego 1A, 10-917 Olsztyn, Poland; ela.lopienska@uwm.edu.pl (E.Ł.-B.); iwona.polak@uwm.edu.pl (I.P.); 3 Department of Plant Physiology, Genetics and Biotechnology, Faculty of Biology and Biotechnology, University of Warmia and Mazury in Olsztyn, Oczapowskiego 1A, 10-719 Olsztyn, Poland; pauk24@gmail.com; 4 Boehringer Ingelheim Vetmedica GmbH, Kathrinenhof Research Center, Walchenseestr. 8-12, 83101 Rohrdorf, Germany; steffen.rehbein@boehringer-ingelheim.com

**Keywords:** *Ostertagia leptospicularis*, morphology, morphometrics, ITS-2, Cervidae, Bovidae, strains

## Abstract

**Simple Summary:**

Pathogenic nematode *Ostertagia leptospicularis*, as the sole member of the subfamily Ostertagiinae, occurs in both cervid and bovid host species. The broad host specificity of this parasite draws special attention and requires a more in-depth investigation. This study was carried out to find out whether the differences in the nematode morphology were only due to the host influence, or whether genetic differences should be taken into account. To resolve this issue, the classification of *O. leptospicularis* was raised and discussed based on its host specificity, as well as morphological and genetic characteristics. A combined morphological–molecular approach was used to compare specimens resembling *O. leptospicularis* collected from naturally infected hosts of various ruminant species (roe deer, red deer, fallow deer, and cattle). Both morphological and molecular analyses highlighted the distinctiveness of *O. leptospicularis* collected from cattle in Germany, and therefore should now be considered to be a different strain that those collected form cervids in central Europe.

**Abstract:**

*Ostertagia leptospicularis* Assadov, 1953 was formally described in roe deer *Capreolus capreolus* and has been reported in a wide range of ruminants, including other Cervidae, as well as Bovidae. Nematode specimens derived from various host species exhibit morphological similarity; however, some differences can be observed. It is unclear if this is due to the differential reaction of one nematode species in different host species (i.e., host-induced changes) or because of distinct nematode species in these hosts (i.e., species complex). This paper focuses on specimens resembling *O. leptospicularis* f. *leptospicularis* and its closely related species (*Ostertagia ostertagi* f. *ostertagi*) collected from various hosts. Morphometric and molecular techniques were applied to assess host-induced changes in nematode morphology and to clarify its systematic classification. There was an overall effect of host species on measurements of nematodes resembling *O. leptospicularis* (both males and females), but the distinctiveness of the specimens from cattle *Bos taurus* were highlighted. The results obtained may suggest that the specimens of *O. leptospicularis* from cattle in Germany and cervids in central Europe belong to different strains. Furthermore, nematodes from the cervid strain appear to circulate within particular host species, which can be seen in the stated morphological variations.

## 1. Introduction 

*Ostertagia leptospicularis* is a member of a group of ruminant stomach worms and, as the sole species of the subfamily Ostertagiinae, seems to be a generalist, capable of infecting a wide variety of hosts [1]. This nematode species was described for the first time by Assadov [2] in roe deer (*Capreolus capreolus*), and therefore, it is considered to be primarily a parasite of Cervidae (red deer (*Cervus elaphus*), sika deer (*C. nippon*), fallow deer (*Dama dama*) and moose (*Alces alces*)); however, it is also able to establish in various representatives of Bovidae (cattle (*Bos taurus*), European bison (*Bison bonasus*), domestic sheep (*Ovis aries*), mouflon (*O. musimon*), domestic goat (*Capra hircus*), Alpine ibex (*C. ibex*), and chamois (*Rupicapra rupicapra*)) [1,3]. 

Some findings indicate that *O. leptospicularis* is highly pathogenic, especially in cattle. It has been proved that its infectivity and pathogenicity in young cattle is higher when compared with blood sucking *Ostertagia ostertagi* (Stiles, 1892) [4]. Moreover, the overall intensity of infection increases if *O. leptospicularis* co-occurs [5,6]. This nematode species may pose a potential threat not only to cattle, but also to other grazed livestock where pastures are shared with feral ruminants treated as principal hosts [7]. Thus, several aspects of the biology of *O. leptospicularis* require further clarification. 

An accurate description of *O. leptospicularis* concerning naturally infected roe deer [2] has been used to identify specimens from other hosts [8,9,10,11,12]. More detailed characterizations, as well as mentions of specimens collected not only from cervids, have emerged thus far; however, they are mainly based on experimental infections [7,13,14,15,16,17,18,19], which interfere with those occurring in natural conditions. As pointed out by Suarez et al. [18], specimens of *O. ostertagi* and *O. leptospicularis* maintained in sheep are of smaller size when compared with those collected from their principal host (cattle and roe deer, respectively). 

Careful study on another species belonging to the subfamily Ostertagiinae, *Teladorsagia circumcincta* (Stadleman, 1894), has revealed morphological analysis to be relevant. Until recently, *T. circumcincta* was treated as a species capable of infecting both domestic and wild ruminant hosts. The most significant research [20,21] demonstrated the presence of two distinct strains within domestic hosts (sheep and goat) that are characterized by morphological and molecular differences. The stated variability in morphology could be attributed to phenotypic plasticity and could correspond to environmental changes (i.e., host species and its resistance to infection) [21]. Such influences have to be considered in this study, especially since accurate identification of ostertagiine nematodes is essential to diagnosis and control of the diseases they cause (i.e., the efficacy of different anthelmintics or its compounds show considerable variations among species) [22]. 

Morphology provides the necessary data to identify a species; however, in the absence of distinguishing features, morphometric methods seem to be more utile. Nevertheless, the remarkable similarity in morphology and morphometrics sometimes makes the identification of individual species doubtful or almost impossible. Although morphology still plays an important role in the species description, taken alone, it often does not provide an adequate taxonomic resolution [23]. Molecular techniques have been proven to be useful for examining the taxonomy of ostertagiine species, where morphological characteristics were unreliable to distinguish between species [20,21,24,25,26]. Many studies have shown that sequences of various regions of ribosomal DNA (rDNA) and mitochondrial DNA (mtDNA) are useful for differentiating morphologically distinct species and detecting the presence of cryptic species that are morphologically similar but genetically distinct [24,25,27,28,29,30,31]. 

Different regions of rDNA (i.e., the intergenic non-transcribed spacer IGS, the external transcribed spacer ETS, transcribed rRNA genes and internal transcribed spacers ITS) evolve at different rates, and hence differ in usefulness for examining encountered problems related to systematics. Sequences of rRNA genes (18S, 5.8S, and 28S) are highly conserved and therefore are of value for examining evolutionary questions [32]. The 18S rRNA gene, due to its stable persistence through generations, is used in phylogenetic analysis among taxa. Sequence variation in this gene within a single species is expected to be very low or absent [33]. Regions of the first and second internal transcribed spacer (ITS-1 and ITS-2, respectively) are less conserved than the genes; however, they are one of the most variable nuclear loci commonly used to discriminate nematode species. ITS-1 and/or ITS-2 have been successfully used in many studies concerning taxonomy of ostertagiine nematodes, because they are highly species-specific [22,25,32,34,35]. ITS-2 in particular has been shown to be a valuable tool for species differentiation. For instance, the lack of difference between ITS-2 of *T. circumcincta*, *T. trifurcata* (Ransom, 1907), and *T. davtiani* Andreev & Satubaldin, 1954 indicated that they represent a single species [36], similar to *Marshallagia marshalli* Ransom, 1907 and *M. occidentalis* Ransom, 1907 [37]. 

Molecular techniques give the opportunity to describe and define biological diversity but are not a panacea for species delimitation. Hence, molecular data are the most useful and valuable when combined with other types of data, especially since morphological characteristics alone are often not adequate for species recognition [38,39]. Thus, synergy between these approaches is needed to collect valid data on biodiversity. An integration of morphological and molecular criteria will have a bearing on understanding the issue [23,24,39]. 

The specific objective of the presented study was to compare specimens resembling *O. leptospicularis* f. *leptospicularis* collected from naturally infected hosts of various ruminant species (roe deer, red deer, fallow deer, and cattle). A combined morphological-molecular approach was used to assess any host-induced changes in nematode morphology and to clarify its systematic classification. Morphological analysis was also undertaken in relation to its closely related species *O. ostertagi* f. *ostertagi* (collected from cattle) to determine, not only intraspecific variations but also interspecific differences. 

## 2. Materials and Methods 

### 2.1. Specimen Collection 

Adult nematodes were derived from abomasum (contents and washings of the mucosa) of naturally infected hosts (*O. leptospicularis*—roe deer, red deer, fallow deer, cattle; *O. ostertagi*—cattle). The origin of hosts (Poland, Slovakia, or Germany) and number of nematode specimens collected and morphometrically analyzed are shown in Table 1. The minor morphs of the species were not included in analysis. 

### 2.2. Morphological Analysis and Measurements 

The examined nematodes (n = 167) were subjected to detailed morphological and morphometric analyses. Entire specimens and/or hand-cut sections were mounted on glass slides in glycerol-jelly or lactophenol [40], photographed, and identified based on morphological features under a zoom-stereomicroscope, and then using a microscope, both equipped with a digital camera. The taxonomy of polymorphic males among the genera and species of abomasal nematodes within the Ostertagiinae follows Dróżdż [41]. 

Morphological characteristics included as measurements were as follows: total body length and esophageal features (both sexes), bursa copulatorix (males), and ovijector and tail (females). Measurements were made for 103 adult specimens of *O. leptospicularis* (59 males, 44 females), as well as 64 adult specimens of *O. ostertagi* (32 each for both males and females) (Table 1, Table 2 and Table 3). Furthermore, the synlophe (i.e., pattern of longitudinal cuticular ridges) was studied in transverse sections for an additional 71 specimens (37 *O. leptospicularis* and 34 *O.* ostertagi; gender participation and host species can be seen in Table 4). 

The anterior and posterior end of the esophageal-intestinal (EI) valves were determined for both sexes based on Lichtenfels et al. [14] and Lichtenfels and Hoberg [42]. The bursa ray pattern and numbering of the bursal papillae were consistent with Dróżdż [8,41] and Jančev [43], as well as Lichtenfels and Hoberg [42]. Hand-cut sections for synlophe identification were made at the EI junction (EIJ) and the midbody for both sexes, as well as anterior to the capitulum of the spicules for males [14,24]. 

### 2.3. Statistical Analyses 

The morphometric data were compared for various host species. Data on worm characteristics (Table 2 and Table 3) were analyzed using multivariate analysis of variance (MANOVA), with *p* < 0.05 being considered statistically significant (Statistica 13 software).

Further analyses included *O. leptospicularis* specimens only and were performed to estimate which variables best separate the host species. All metrical features (variables) included in Table 2 were submitted to discriminant analysis using the host species as a grouping variable (Statgraphics Centurion 18 software). Specimens with a missing value were not included in the canonical analysis. The forward stepwise variable selection procedure was applied using the minimization of Wilks’ lambda as a selection criterion.

### 2.4. Molecular and Phylogenetic Analyses 

Total genomic DNA was extracted from the midbody of specimens used in morphological analyses (*O. leptospicularis* from roe deer, red deer, and fallow deer) or pooled third-stage larvae (*O. leptospicularis* from cattle) using Sherlock AX (A&A Biotechnology, Poland), according to the manufacturer’s instructions. The midbody of the specimens was cut after species identification and measuring the total length. Extracted DNA was stored at −20 °C for subsequent analysis. 

The second internal transcribed spacer (ITS-2) of the ribosomal DNA (rDNA) was used as the target to define specific marker. ITS-2 was amplified using forward and reverse primers (5′-CAGACGCTTAGAGTGGTGAA-3′ and 5′-TGCATGTGTTTTCTTGAACTGA-3′, respectively) designed from the aligned sequences available in GenBank (Geneious software 13.0). The polymerase chain reaction (PCR) mix was comprised of 0.5-unit Platinum Taq polymerase (LifeTechnologies), 1xTaq buffer, 2.5 mM MgCl_2_, 200 µM each dNTP, 20 pmol of each primer, and 4 µL genomic DNA. 

The PCR conditions were as follows: a single step of pre-denaturation at 94 °C for 8 min (min), followed by 35 cycles of 94 °C for 30 s (sec), 55 °C for 30 s, 72 °C for 1 min, and the final extension at 72 °C for 8 min. PCR products were confirmed by gel electrophoresis in 2% agarose gels and visualized with Midori Green Advance (Polgen) staining. The purified PCR products were sequenced (Genomed, Poland) and compared with sequences available in the GenBank database using Geneious Prime v. 11.0.610 [44]. 

New nucleic acid sequences (ten shared by *O. leptospicularis* derived from various host species) were deposited in GenBank.

The newly obtained sequences of ITS-2 rDNA were aligned in MAFFT v7.310 with a selected set of other nematode sequences (including *Ostertagiinae* sp.) downloaded from the NCBI database, with *Haemonchus contortus* used as an outgroup. Bayesian Inference (BI) was used for genome-wide phylogenetic analyses in MrBayes v.3.2.6 [45]. Before BI analysis, the best fitting substitution model was searched in Mega 7 [46], and the model JC69 + G was selected. BI partitioning analysis was carried out to develop a majority rule consensus tree with 1 × 10^6^ generation using the Markov Chain Monte Carlo (MCMC) method. The tree sampling frequency was 200 generations. 

## 3. Results 

### 3.1. Morphological and Morphometric Analyses 

**Figure 1:**
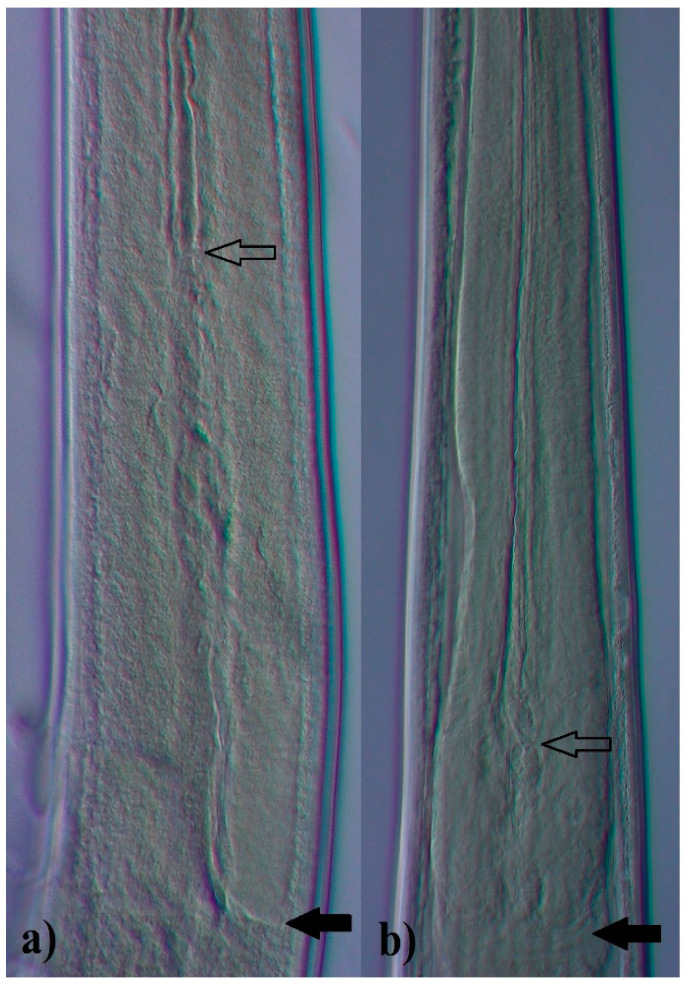
Esophageal-intestinal (EI) valve of (**a**) *Ostertagia leptospicularis* from roe deer (*Capreolus capreolus*), (**b**) *Ostertagia ostertagi* from cattle (*Bos taurus*; arrows at anterior and posterior end; magnification 600×).

**Figure 2:**
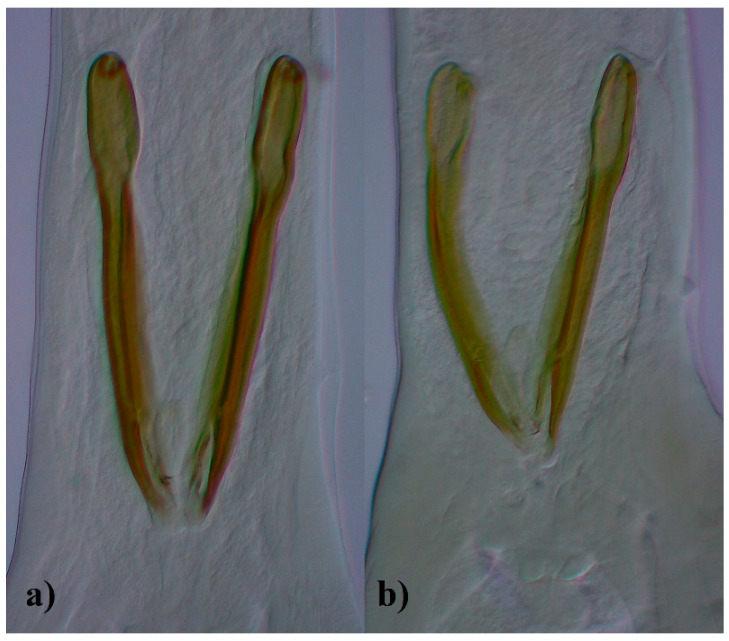
Spicules of *Ostertagia leptospicularis* from (**a**) roe deer (*Capreolus capreolus*), (**b**) cattle (*Bos taurus*) ventral view (magnification 400×).

**Figure 3:**
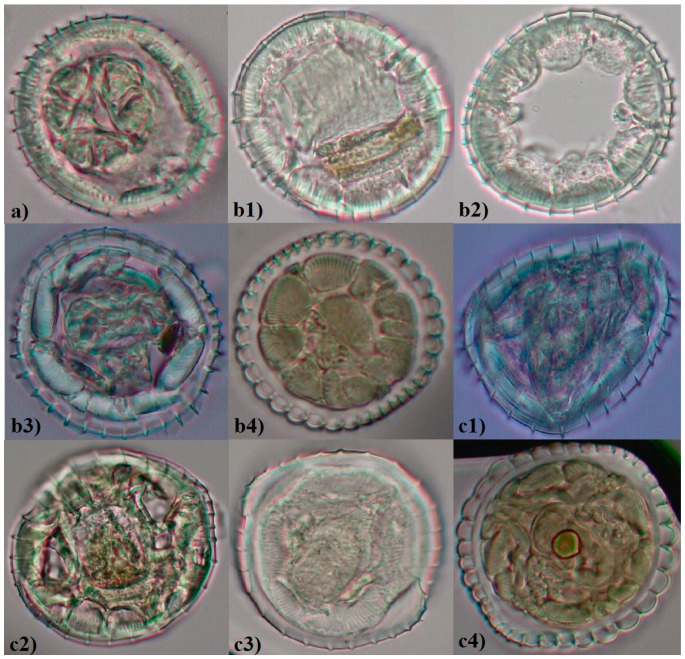
Longitudinal cuticular ridges of *Ostertagia leptospicularis* males shown in transverse sections (**a**) at the esophageal-intestinal (EI) valves, n = 36, host roe deer (*Capreolus capreolus*); (**b1–4**) at the midbody: (**b1**) n = 31, cattle (*Bos taurus*); (**b2**) n = 32, cattle; (**b3**) n = 35, red deer (*Cervus elaphus*); (**b4**) n = 36, roe deer; (**c1–4**) above the anterior end of the spicules: (**c1**) n = 30, red deer; (**c2**) n = 31, cattle; (**c3**) n = 32, roe deer; (**c4**) n = 34, roe deer (magnification 600×).

The analyzed nematodes, both males and females, exhibited some morphological similarity. The characteristic that easily distinguished the studied species was the esophagus, which was clearly visible, but differed with respect to the length of EI valves. Prominent long EI valves characterized nematodes from *O. leptospicularis*, while explicitly short valves were those from *O. ostertagi* (Figure 1). Cuticular ridges were stated in all specimens but differed in number in relation to the nematode and host species. Nevertheless, the number of ridges in each case attained the highest value at the EIJ and decreased towards the posterior end of the body. The bursa ray formula 2-1-2 was present in both species studied herein. Furthermore, the spicules of *O. leptospicularis* from its principal host (roe deer) clearly showed differences when compared with those obtained from cattle (Figure 2). Characteristics and measurements of *O. leptospicularis* and *O. ostertagi* (both males and females) are noted in Table 2 and Table 3, respectively, and the observed differences are listed below. The number of longitudinal ridges for additionally analyzed specimens are shown in Table 4 and Figure 3. 

#### 3.1.1. Male Specimens

There was an overall diversity of nematodes resembling *O. leptospicularis* collected from different host species (Table 2). There were significant differences between measurements concerning the length of the (i) body, (ii) EI valves, and (iii) both spicules, as well as (iv) EI valve width. Most differences were noted between specimens collected from Bovidae and Cervidae; however, differences among representatives of Cervidae also emerged. No significant differences were observed between measurements of total esophagus length or trifurcation of the spicules. 

**Figure 4:**
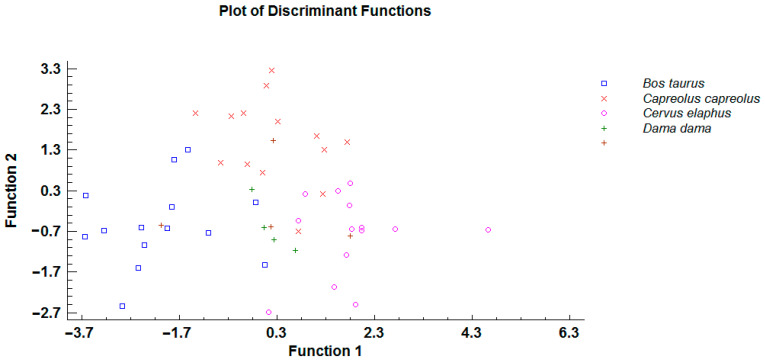
Scatter plot of discriminant function analysis comparing selected morphological characteristics of *Ostertagia leptospicularis* males derived from different host species (brown crosses indicates particular centroides).

Using a forward stepwise selection algorithm, it was determined that four variables were significant predictors of host species (total body length, intestinal valve length and width, left spicule length). Both canonical discriminant functions had small values of Wilks’ lambda (0.126165 and 0.4477, respectively) and were significant (Chi-squared approximation, *p <* 0.0001). As illustrated in Figure 4, the first function separated specimens from cattle and cervid host species, and the second function differentiated specimens from principal host (i.e., roe deer) and other host species. 

All specimens studied had a similar number of longitudinal ridges over many of the analyzed levels (Table 4). Most of the studied specimens had 36 longitudinal ridges at the EIJ, excluding those collected from roe deer (in some cases it was 35 ridges). The number of longitudinal ridges at the midbody ranged from 29 to 36 and specimens derived from cattle varied the most. Slightly reduced variability (i.e., 29–34 ridges) was noted just above the anterior end of the spicules. No differences were observed between specimens collected from red deer and cattle, while those form roe deer at this level varied the most. 

No significant differences were found between *O. ostertagi* derived from cattle differing in the country of origin (Table 3). 

Transverse sections were possible to be made for *O. ostertagi* from Germany only, hence the comparison of longitudinal ridges between specimens differing in the country of origin could not be made. The lowest/highest number of ridges at all studied levels were lower/higher, respectively, when compared with those obtained for the specimens of *O. leptospicularis* (excluding the highest value reached just above the spicules) (Table 4). 

#### 3.1.2. Female Specimens

There were significant differences between all measurements made for *O. leptospicularis* from different hosts (Table 2). Most of the differences, similar to males, were between Bovidae and Cervidae; however, the differences among representatives of Cervidae have also been stated. 

**Figure 5:**
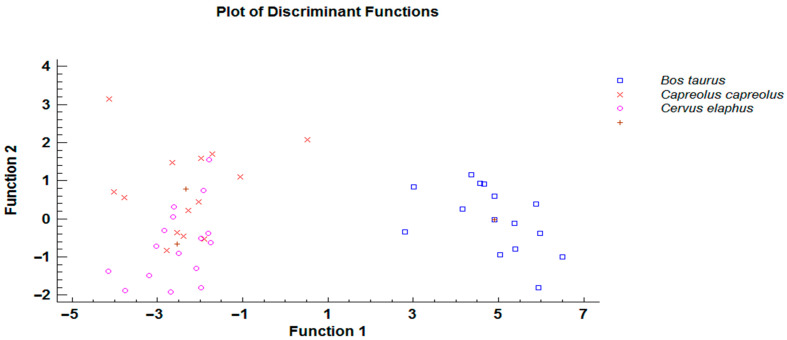
Scatter plot of discriminant function analysis comparing selected morphological characteristics of *Ostertagia leptospicularis* females derived from different host species (brown crosses indicates particular centroides).

Using a forward stepwise selection algorithm, it was determined that four variables were significant predictors of host species (intestinal valve length and width, ovijector length and tail length). Both canonical discriminant functions had small values of Wilks’ lambda (0.0528644 and 0.732462, respectively) and were significant (Chi-squared approximation, *p <* 0.0001). As illustrated in Figure 5, the first function clearly separated specimens from cattle and cervid host species.

Slightly smaller differences in the number of longitudinal ridges were observed between *O. leptospicularis* specimens derived from different ruminant hosts; however, those from roe deer proved to be slightly more diverse than those from cattle (Table 4). 

Significant differences were found between measurements made for *O. ostertagi* from cattle of Polish and German origins, concerning the length of the (i) body, (ii) esophagus, and (iii) ovijector, as well as (iv) EI valve width (Table 3) 

Transverse sections were not made for *O. ostertagi* from Poland, hence the comparison with those conducted for specimens from Germany could not be made. The largest differences were observed at the midbody, where the lowest value reached for *O. ostertagi* was quite like the highest for *O. leptospicularis* (Table 4). 

### 3.2. Molecular and Phylogenetic Analyses 

**Figure 6:**
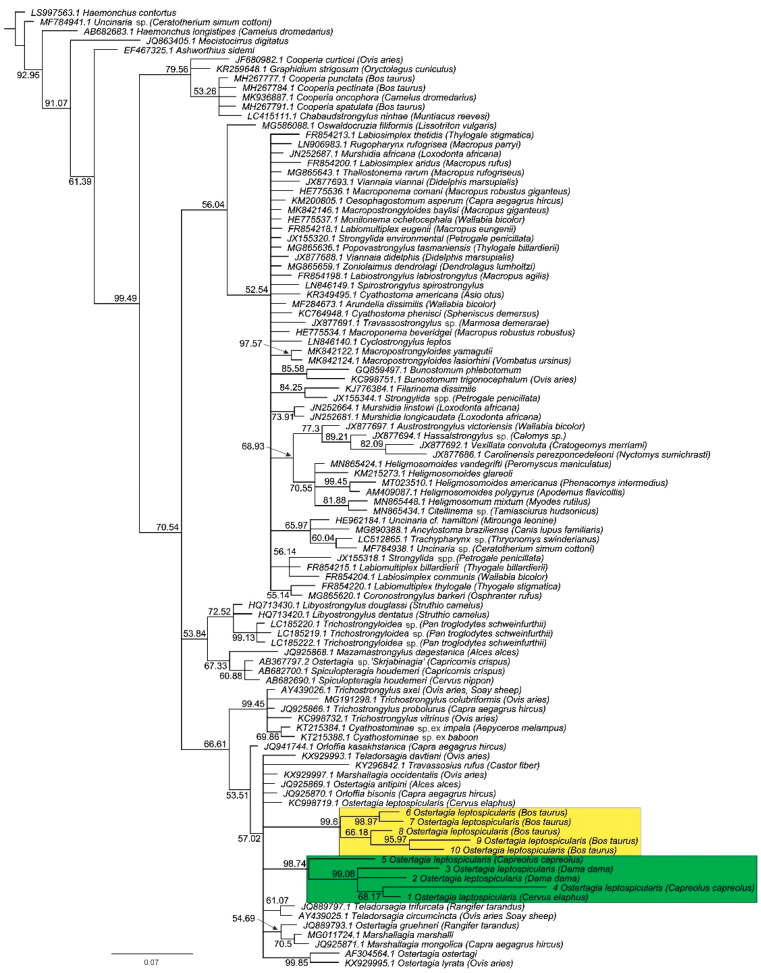
Consensus tree depicting the ITS-2 similarity of newly obtained sequences of *Ostertagia leptospicularis* (1–MT954614 221bp, 2–MT954616 219bp, 3–MT954631 219bp, 4–MT956640 225bp, 5–MT954907 222bp, 6–MT956639 145 bp, 7–MT954410 150bp, 8–MT954415 149bp, 9–MT954592 152bp, 10–MT956639 145bp) and those available in the NCBI database. Bayesian posterior probabilities (PP) are given at each node.

The phylogenetic tree (Figure 6) represents the similarity level among ITS-2 rDNA sequences of the analyzed samples (numbers from 1 to 10 before the nematode species name; see Figure 6 legend for GenBank accession numbers of newly obtained sequences) and other nematode species. The location of the studied sequences in the presented tree at a considerable distance from each other, when isolated from the same or close phylogenetically host species, could be the basis for inferring the randomness of the formation of separate populations. Our previous study [1] showed that nematodes of the subfamily Ostertagiinae appear to be rather specific to a species or family of hosts. Thus, the presented phylogenetic tree was extended to other Ostertagiinae species and their hosts, and it showed that *O. leptospicularis* form separate populations specialized in colonizing one type of host.

Within the studied specimens, it was possible to clearly mark two clusters in the phylogenetic tree with *O. leptospicularis* derived from cattle (marked in yellow) and cervid host species (marked in green). Nematode specimens from both clusters were characterized by high levels of intraspecific variability in the ITS-2 rDNA sequences. This may indicate that nematodes circulate within particular host species (i.e., roe deer, red deer, and fallow deer).

## 4. Discussion 

*Ostertagia leptospicularis* was originally described in roe deer from Azerbaijan [2]; however, its widespread occurrence suggests an extensive historical and geographical range. This species has been treated, so far, as a typical parasite of cervids and domestic bovids in the Palearctic region [8,13,47], although it has been successfully introduced to other Holarctic regions and identified in many host species [1,17]. 

The results obtained herein appear to be partially consistent with the first description of *O. leptospicularis.* The data concerning morphometric criteria are not so numerous, but it is difficult to disagree with Kutzer and Hinaidy [47], who analyzed *O. leptospicularis* from wild ruminants (roe deer, red deer, chamois) in Austria and suggested that Assadov [2] measured atypical specimens. The range of the body and esophagus length presented in the first description was higher than the mean values attained in this study. The results presented by Assadov [2] are most like those obtained for specimens derived from red deer. However, the measurements of EI valve width and spicule length, made for specimens collected from roe deer, are consistent with the first ones. A comparison of these values depicts the nematodes analyzed the first time as having wider EI valves and longer spicules. The latter ones attract special attention due to the high minimum value, which pleads in favor of atypicality of specimens described by Assadov [2]. As pointed out in some studies, spicule length is a morphological feature that distinguishes individuals from roe deer and red deer. This research not only confirmed that those from red deer are slightly smaller [43,47,48], but also proved that *O. leptospicularis* from fallow deer and cattle have even shorter spicules. Nevertheless, the outcomes obtained herein suggest that based on the length of spicules, the specimens from different cervid host species seem to remain undistinguished, while those from cattle are outstanding. The same conclusion arises from outcomes concerning body length. 

As demonstrated by Lichtenfels et al. [14] and Lichtenfels and Hoberg [42], differences not only in spicules and genital cones, but also in the length of EI valves, are useful for identifying males of Ostertagiinae to the species level. This morphological feature of *O. leptospicularis* was included in only a few analyses (i) made in North America, (ii) based on artificial infections, (iii) presented outcomes for *O. leptospicularis* along with those for *O. kolchida*, and/or (iv) with combined results obtained for specimens collected from bovids (cattle, sheep and goat) [9,14,17]; hence, comparable data cannot be gathered. Most available papers concerning the morphology of *O. leptospicularis* are limited to those that give the values of length and/or width of the body, esophagus, spicules, and/or gubernaculum [13,18,43,47,48,49]. The length of EI valves is nonetheless a perfect criterion to distinguish *O. leptospicularis* (both males and females) from *Ostertagia ostertagi*, as well as other closely related representatives of the subfamily Ostertagiinae. The identification of females of most ostertagiine species has so far been treated as impossible to conduct in mixed infections [7]; however, the length of the mentioned structure is easy to observe and has distinctly simplified the issue. Such a conclusion has been already suggested by Lichtenfels and Pilitt [50]. As demonstrated in this research, regardless of host species, *O. leptospicularis* had EI valves that were almost twice as long as *O. ostertagi* (both sexes) (Figure 1). Moreover, *O. leptospicularis* derived from cattle were noted once more as having become an outstanding species, due to its prominently long EI valves. 

Lichtenfels et al. [7,14] revealed that differences in the length of EI valves were corelated with differences observed in the lateral synlophe. Species with a Type 2 lateral synlophe (i.e., three continuous and parallel ridges) had a long valve, while those with a Type 1 (i.e., only one continuous ridge in each lateral field) had a shorter valve. Other studies of the Ostertagiinae of domestic and sylvatic hosts have demonstrated the utility of the synlophe as a key in separating species [50,51,52]. Nonetheless, papers concerning the number of cuticular ridges of *O. leptospicularis* are limited, and are focused mainly on those at the midbody [13,18,19], which has been revealed herein as the most variable. This result is not consistent with further findings that suggest the number of ridges at the midbody to be constant. In the present research, there was enough variability to disqualify the synlophe as a diagnostic characteristic. The number of cuticular ridges may be useful, although only as a supplementary feature to the length of the EI valves. 

In this study, variability in some measurements of adult *O. leptospicularis* from different host species was detected. A comparison of male measurements depicted those from (i) roe deer as having the longest esophagus, (ii) cattle as having the longest and widest EI valves, and (iii) roe deer as having a slightly longer left and right spicule when compared with other hosts. Nevertheless, spicules of specimens derived from cattle appeared to be strikingly smaller than those from cervid hosts (Figure 2). A comparison of females revealed that those from cattle had the highest values of the majority of analyzed characteristics, except for the total ovijector length. The majority of the aforementioned differences may be treated as host-induced ones; however, the distinctiveness of *O. leptospicularis* from cattle should be highlighted. 

Such a conclusion is consistent with the results of genetic analyses, in which *O. leptospicularis* collected from cattle in Germany were clearly separated from those derived from cervid host species in central Europe (Figure 6). Therefore, it can be suggested that the specimens from *Bos taurus* in Germany and cervid host species in central Europe belong to different strains. Furthermore, nematodes from the latter strain circulate within particular host species that can be seen in the morphological variations of specimens derived from roe deer, red deer, and fallow deer. It is also consistent with the results of our prior study where roe deer were identified to be a principal host of *O. leptospicularis* [1]. The aforementioned circulation may also exist among bovid host species, which requires further analyses, enriched with a wider molecular approach. 

## 5. Conclusions

The morphological features that would allow us to consider the analyzed nematodes as different species have not yet been stated. Whether *O. leptospicularis* from cattle in Germany and Cervidae in central Europe should be treated as different strains or lines (strongly connected with the host species) able to circulate among hosts from a particular family requires further investigation. This is important due to the possibility of parasite exchange in the pastures shared by domestic and wild ruminants, as well as a higher probability of this nematode species being introduced by imported bovids. To date, *O. leptospicularis* has been identified in cattle from only a few European countries, including Germany, Austria, Belgium, and the Netherlands [53,54,55,56,57], and therefore even more attention should be dedicated to this species.

Although the Ostertagiinae subfamily is the subject of consistent study, many issues are still uncertain. New information on *O. leptospicularis* presented herein shows that some other findings are required to develop an identification key to both males and females of this species (e.g., details of the esophagus, genital cone and synlophe). It is apparent that more characteristics and nematode specimens resembling *O. leptospicularis* from all possible hosts have to be studied before any revision of the systematics can be made. More valid conclusions must await the results of other studies that are currently in progress.

## Figures and Tables

**Table 1:** Origin of hosts and number of nematode specimens morphometrically analyzed.

Host		*Ostertagia leptospicularis*		*Ostertagia ostertagi*	
		Males	Females	Males	Females
roe deer *Capreolus capreolus*	Poland/ Slovakia	20	14	- -	- -
red deer *Cervus elaphus*	Poland	15	16	-	-
fallow deer *Dama dama*	Poland	4	-	-	-
Cattle *Bos taurus*	Poland Germany	- 20	- 14	12 20	16 16

**Table 2:** Measurements of *Ostertagia leptospicularis* derived from different host species (presented in micrometers unless indicated otherwise and given as a range followed by the mean ± SD in parentheses; values in rows with the same letters differ significantly).

Characteristics of Nematode Specimens		Male Specimens Derived from				Female Specimens Derived from		
		Roe Deer *Capreolus capreolus*	Red Deer *Cervus elaphus*	Fallow Deer *Dama ama*	Cattle *Bos taurus*	Roe Deer *Capreolus capreolus*	Red Deer *Cervus elaphus*	Cattle *Bos taurus*
		n = 20	n = 15	n = 4	n = 20	n = 14	n = 16	n = 14
Body	Total length	5984.4–7485.2 (6684.4 ± 447.3)	6245.3–7888.7 (7063.3 ^A^ ± 523.1)	6905–7425.5 (7144.1 ± 216.8)	5905.9–6976.1 (6519.8 ^A^ ± 326.1)	7752–11,736.6 (9478.8 ^A^ ± 1051.1)	7374.2–9038.1 (8187.1 ^AB^ ± 563.1)	9259.4–12,108.4 (10,436.6 ^B^ ± 793.8)
Esophagus	Total length	707.5–803.6 (772.1 ± 29.3)	705.2–898.9, (766.7 ± 51.3)	711.8–812.3 (762.9 ± 44.5)	645.1–818.4 (764.9 ± 43.5)	699.7–848.8 (788.8 ^A^ ± 40.2)	705.5–819.6 (766.2 ^B^ ± 36)	765.9–908.9 (847.9 ^AB^ ± 43.5)
	Intestinal valve length	117.5–168.4 (133 ^A^ ± 12.7)	101.9–131.4 (111.8 ^AB^ ± 7.8)	119.8–127.5 (123.8 ± 3.3)	111.4–176.4 (134.8 ^B^ ± 19.6)	103.9–133.5 (115.6 ^A^ ± 9.4)	101.6–124.5 (115.4 ^B^ ± 7.1)	131.7–176 (157 ^AB^ ± 13.4)
	Intestinal valve width	22.4–53 (34.4 ^A^ ± 9)	15.8–32.5 (25.2 ^AB^ ± 4)	29.4–35.9 (33.2 ± 2.8)	28.1–46.9 (39 ^B^ ± 6.1)	23.1–37 (29.9 ^A^ ± 4.2)	23.1–34 (28.5 ^B^ ± 3)	34.9–52.4 (43.1 ^AB^ ± 5)
Bursa copulatorix	Spicule left (length)	163.9–209.1 (183.3 ^A^ ± 11.3)	166.4–169.5 (178.7 ^B^ ± 9.1)	177.2–182.3 (179.4 ± 2.3)	148.6–177.5 (165.1 ^AB^ ± 8.2)	-	-	-
	Trifurcation (length)	32.3–48.5 (40.4 ± 4.5)	35.7–45.5 (40.4 ± 2.6)	40.1–42.4 (41.3 ± 1.1)	35.1–45.2 (40.5 ± 2.6)	-	-	-
	Spicule left (% trifurcation)	75.3–82.1 (78 ± 1.6)	75.4–79.6 (77.4 ± 1.2)	76.2–78 (77 ± 0.8)	73.2–78.4 (75.5 ± 1.4)	-	-	-
	Spicule right (length)	163.2–205.4 (178.6 ^A^ ± 12.1)	161.9–193.8 (174.3 ^B^ ± 9.5)	170.4–177.8 (174 ± 3.6)	143–172.3 (159.6 ^AB^ ± 8.8)	-	-	-
	Trifurcation (length)	29.6–49.7 (35.5 ± 4.9)	25.6–39.7 (33.8 ± 3.8)	33.4–38.4 (36.5 ± 2.1)	29.3–37.8 (33.9 ± 2.7)	-	-	-
	Spicule right (% trifurcation)	75.8–88.8 (80.2 ± 1.7)	78.7–84.7 (80.6 ± 1.6)	78.4–80.4 (79.1 ± 0.9)	76.3–81.6 (78.9 ± 2.7)	-	-	-
	Sjöberg’s organ	absent	absent	absent	absent	-	-	-
	Bursal ray pattern	2-1-2	2-1-2	2-1-2	2-1-2	-	-	-
Ovijector	Total length	-	-	-	-	172.8–282.1 (207.8 ^AB^ ± 27.7)	158.4–228.7 (181.7 ^B^ ± 18)	113.7–193.2 (164.3 ^A^ ± 19)
Tail	Total length	-	-	-	-	145.1–183.2 (160.8 ^Ab^ ± 13.4)	132.7–162.8 (149.8 ^bC^ ± 10.8)	179.7–207.3 (190.8 ^AC^ ± 9.5)

A, B, C values in rows with the same letters differ significantly at *p* < 0.01. b values in rows with the same letters differ significantly at *p* < 0.05.

**Table 3:** Measurements of *Ostertagia ostertagi* derived from cattle (*Bos taurus*)—its principal host (presented in micrometers unless indicated otherwise and given as a range followed by the mean ± SD in parentheses; values in rows with the same letters differ significantly at *p* < 0.01).

Characteristics of Nematode Specimens		Male Specimens		Female Specimens	
		n = 12 *	n = 20 **	n = 16 *	n = 16 **
Body	Total length	5010.3–7701.3 (6567.9 ± 795.6)	5827.9–7890.6 (6867.3 ± 573.5)	9834.8–11,083.5 (10,349 ^A^ ± 389.5)	8676.9–11,236.1 (9753.4 ^A^ ± 615.1)
Esophagus	Total length	575.6–710.1 (645.3 ± 40)	580.4–727.4 (649.3 ± 34.1)	654–766.3 (726.3 ^A^ ± 27.7)	642.2–745.5 (675.8 ^A^ ± 30.6)
	Intestinal valve length	50.9–67.8 (61.9 ± 5.1)	51.1–67.7 (58.3 ± 5.4)	54.6–82.3 (68.6 ± 10)	56.7–85.8 (66.1 ± 7.5)
	Intestinal valve width	33.5–58 (45 ± 8.8)	35.5–69.9 (47.1 ± 9)	40.1–64.3 (52.1 ^A^ ± 6)	47.7–71.4 (60.2 ^A^ ± 5.9)
Bursa copulatorix	Spicule left (length)	222.9–243 (233.6 ± 7)	215.8–249.6 (229 ± 7.7)	-	-
	Trifurcation (length)	50.8–63.1 (57.5 ± 3.1)	51.9–62.5 (57.8 ± 3.2)	-	-
	Spicule left (% trifurcation)	73.5–77.2 (75.4 ± 1)	73.3–77 (74.8 ± 1.2)	-	-
	Spicule right (length)	216.9–242.2 (232 ± 8.1)	215.3–249.4 (227.6 ± 7.5)	-	-
	Trifurcation (length)	51.1–65.1 (58.9 ± 3.5)	54.2–65.3 (59.3 ± 3.3)	-	-
	Spicule right (% trifurcation)	72.2–76.9 (74.6 ± 1.2)	72–76.1 (74 ± 1.3)	-	-
	Sjöberg’s organ	absent	absent	-	-
	Bursal ray pattern	2-1-2	2-1-2	-	-
Ovijector	Total length	-	-	224.9–318 (273.1 ^A^ ± 23.4)	219.1–274.5 (247.6 ^A^ ± 17.2)
Tail	Total length	-	-	119.6–159.7 (138.5 ± 13.1)	121.9–154.4 (141.5 ± 9.8)

* origin: Poland; ** origin: Germany.

**Table 4:** Number of longitudinal cuticular ridges of nematodes in relation to host species.

Characteristics of Nematode Specimens		*Ostertagia leptospicularis*					*Ostertagia ostertagi* *	
		Male Specimens (n = 27) Derived from			Female Specimens (n = 10) Derived from		Male SpecimensDerived from	Female Specimens Derived from
		Roe Deer *Capreolus capreolus*	Red Deer *Cervus elaphus*	Cattle *Bos taurus*	Roe Deer *Capreolus capreolus*	Cattle *Bos taurus*	Cattle *Bos taurus*	
		n = 10	n = 8	n = 9	n = 5	n = 5	n = 21	n = 13
Cuticular ridges	esophageal-intestinal junction (EIJ)	35–36	36	36	35–36	36	33–40	34–39
	midbody	32–35	32–36	29–34	29–32	29–30	33–39	30–36
	spicules	30–34	29–32	29–32	-	-	26–31	-

* origin: Germany.

## Data Availability

The data supporting the conclusions of this article are included within the article. The raw data used and/or analyzed during the current study are available from the corresponding author upon reasonable request.
